# Utilizing Centromedian Thalamus Connectivity to Personalize Noninvasive Neuromodulation Targets

**DOI:** 10.1111/cns.70120

**Published:** 2024-12-08

**Authors:** Cong Fu, Zijian Feng, Qiu Ge, Juan Yue, Yu‐Feng Zang, Guoming Luan

**Affiliations:** ^1^ Department of Neurosurgery, Epilepsy Center, Sanbo Brain Hospital Capital Medical University Beijing China; ^2^ Epilepsy Institution Beijing Institute of Brain Disorders Beijing China; ^3^ Transcranial Magnetic Stimulation Center Deqing Hospital of Hangzhou Normal University Deqing Zhejiang China; ^4^ Methods and Development Group “Brain Networks” Max Planck Institute for Human Cognitive and Brain Sciences Leipzig Germany; ^5^ Lise Meitner Research Group "Cognition and Plasticity" Max Planck Institute for Human Cognitive and Brain Sciences Leipzig Germany; ^6^ Center for Cognition and Brain Disorders The Affiliated Hospital of Hangzhou Normal University Hangzhou Zhejiang China; ^7^ Zhejiang Key Laboratory for Research in Assessment of Cognitive Impairments Hangzhou Zhejiang China; ^8^ Institute of Psychological Sciences Hangzhou Normal University Hangzhou Zhejiang China; ^9^ Laboratory for Clinical Medicine Capital Medical University Beijing China

**Keywords:** centromedian nucleus of the thalamus, functional connectivity, resting‐state fMRI, transcranial magnetic stimulation

## Abstract

**Introduction:**

The centromedian nucleus (CM) of the thalamus is essential for arousal, attention, sensory processing, and motor control. Neuromodulation targeting CM dysfunction has shown efficacy in various neurological disorders. However, its individualized precise transcranial magnetic stimulation (TMS) remains unreported. Using resting‐state functional MRI, we mapped CM‐based functional connectivity (CM‐FC) to develop a personalized TMS scheme for neurological conditions.

**Methods:**

We first analyzed the CM‐FC patterns of healthy subjects via 10 scanning sessions in three MRI scanners spanning two subject groups: one from the Human Connectome Project (*n* = 20, four sessions) dataset and the other from Hangzhou Normal University (*n* = 20, three sessions of 3 T MRI and three sessions of 1.5 T MRI). Pearson's correlation was used for CM‐FC evaluation. Then, we proposed an overlapping index ranging from 1 to 10, and group‐level clusters with the highest overlapping index located 4 cm beneath the scalp were identified. In the individual CM‐FC map, watershed image segmentation was used to obtain an individual cluster. The peak voxel with the highest FC value within the individual cluster was defined as a potential individualized target for future TMS.

**Results:**

The spatial FC patterns were remarkably similar between the left and right CMs. CMs have widespread positive connectivity with cortical areas, including the sensorimotor cortex, supplementary motor area, middle frontal cortex, medial temporal cortex, and middle cingulate. Among the group‐level FC patterns of the left and right CMs, only the left CM had a group cluster in the left primary sensorimotor cortex (PSMC, cluster size = 51) with an overlapping index of 10, that is, 10 sessions showed significant CM‐FC.

**Conclusions:**

The left PSMC exhibited reproducible FC with the left CM. The individual peak FC location in the left PSMC could be used as a TMS target for indirect modulation of CM activity and aid in the treatment of CM‐related neurological disorders.

## Graphical abstract

The authors propose an approach for CM‐targeting individual TMS, using two group clusters of left and right CM which were functionally connected with the left primary sensorimotor cortex (PSMC) and the right superior of frontal gyrus (SFG) in the RS‐fMRI analysis.

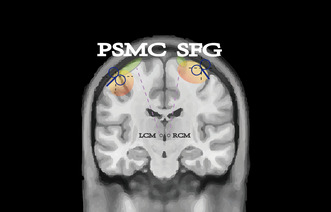

## 1 Introduction

The centromedian nucleus (CM) is a part of the intralaminar thalamic nucleus and is involved in relaying sensory and motor information to and from the cerebral cortex. The CM is situated within the central region of the thalamus, and its connections extend to various brain regions, including the basal ganglia, limbic system, and cerebral cortex. This extensive network of connections suggests that the CM is involved in a wide range of cognitive, motor, and emotional processes [1]. This nucleus is known to play a crucial role in various neurological functions, and its dysfunction has been associated with a range of neurological disorders, such as refractory generalized epilepsy [2], disorders of consciousness [3], Tourette syndrome [4], and neuropathic pain [5]. Many clinical studies have used the CM as a direct stimulation target for invasive neuromodulation treatments, such as deep brain stimulation (DBS) [6, 7, 8] while no studies have investigated CM as an effective target for noninvasive neuromodulation, such as transcranial magnetic stimulation (TMS).

Invasive DBS procedures based on neural circuit mechanisms have long been used to treat functional disorders. However, the risk of surgical complications and the high cost of implantation equipment are burdensome for many patients and their families. As a widely used noninvasive neuromodulation tool, TMS has been shown to improve symptoms and quality of life in patients with various conditions in many clinical trials and meta‐analyses [9]. In detail, TMS has been used for antiepileptic treatment, and many studies have reported that TMS can be used to achieve seizure control for patients with refractory generalized epilepsy [10, 11] as well as potential benefits in patients with disorders of consciousness [12, 13] and children with Tourette syndrome [14]. TMS has also been approved by the Food and Drug Administration (FDA) and other regulatory agencies for some of these indications. Therefore, TMS may be an alternative tool or a prior effect test method for invasive neuromodulation approaches.

Previous studies indicate that TMS neuromodulation induces changes in resting‐state functional connectivity (RSFC) [15, 16]. Since the CM can be used as a target in DBS neuromodulation, it could also be regarded as an effective target for TMS because the cortical locations are nodes in the same brain network. Moreover, many studies have indicated that resting‐state functional magnetic resonance imaging (RS‐fMRI)‐derived RSFC could guide TMS targeting individually and precisely [17, 18, 19]. Therefore, using CM‐based RSFC (CM‐FC) for TMS neuromodulation may have the potential to improve clinical manifestations in patients treated with CM‐DBS. Although the spatial pattern of CM‐FC has been reported [20], the site of TMS intervention for future individualized TMS therapy needs to be considered. However, no study has reported the associated area or region for TMS neuromodulation because of extensive CM‐FC mapping.

Overall, the goals of this study were to identify the CM‐FC spatial pattern and identify two feasible cortical clusters of the left and right CMs for individual practice. Here, we used a watershed segmentation method on functional images to achieve a subject‐specific target for TMS. We hope this study will advance our understanding and improve the further application of individual TMS in CM modulation.

## 2 Materials and Methods

### 2.1 Data Acquisition

In this paper, we studied the intersubject generality of RS‐fMRI‐guided targets using three MRI scanners from the Human Connectome Project (HCP, *n* = 20) and Hangzhou Normal University (*n* = 20), including a 3.0 T MRI scanner from the Center of Cognition and Brain Disorders (CCBD) and a 1.5 T MRI scanner from the TMS Center of Deqing Hospital (TCDQ). The CCBD and TCDQ datasets were obtained from the same healthy subjects scanned by different scanners. We collected imaging data from 10 scanning sessions: four sessions from the HCP and three sessions each from the CCBD and TCDQ. The details are provided in Table 1.

**TABLE 1:** Resting‐state fMRI scanning parameters from the three MRI scanners.

Dataset	Number of subjects	Field strength (Tesla)	Manufacturer and model	Session	TR/TE (ms)	Scan length	Volumes	Voxel size (mm^3^)	Eyes open
HCP	20	3.00	Siemens Skyra	4	720/33	15′00″	1200	2.0 × 2.0 × 2.0	4 (+)
CCBD	20	3.00	GE MR750	3	700/30	8′10″	700	3.0 × 3.0 × 3.0	2 (+) 1 (−)
TCDQ	20	1.57	UI uMR588	3	3000/40	8′30″	180	3.5 × 3.5 × 3.5	2 (+) 1 (−)

*Note:* CCBD and TCDQ datasets were collected from the same cohort of participants. (+) eyes open; (−) eyes closed.

Abbreviations: CCBD, Center of Cognition and Brain Disorders; HCP, Human Connectome Project; TCDQ, TMS Center of Deqing Hospital.

#### 2.1.1 HCP dataset

We analyzed the CM‐FC pattern using MRI data collected with a 32‐channel head coil from 20 subjects in an HCP study. Images of four 15‐min RS‐fMRI sessions were obtained per subject on two different days. Scans were repeated twice using different phase encoding directions (from left to right and from right to left) each day. Participants were asked to keep their eyes open and focus on a fixation point during the RS‐fMRI scan.

#### 2.1.2 CCBD and TCDQ datasets

As mentioned above, these two datasets were collected from the same cohort of participants (*n* = 20). Each of the 20 participants underwent six RS‐fMRI sessions on one day, with three sessions per dataset. They were scanned three times in a 3 T scanner equipped with a 48‐channel head coil and a 1.57 T scanner equipped with a 16‐channel head coil. For CCBD RS‐fMRI image acquisition, a multiband gradient echo‐planar imaging (GRE‐EPI) sequence was used, and a single‐shot GRE‐EPI sequence was used for the TCDQ dataset. The order of 3 T and 1.57 T scanning was counterbalanced, and the mean inter‐scanner interval was 3.87 ± 0.8 h. RS‐fMRI included an eyes‐open session (EO1), a second eyes‐open session (EO2), and an eyes‐closed session (EC). The order of the EO and EC sessions was counterbalanced across participants. Participants were asked to either keep their eyes closed or to keep their eyes open and to not think about anything in particular during the RS‐fMRI scan. The CCBD and TCDQ ethics committees approved the study. All participants signed written informed consent prior to scanning.

### 2.2 Resting‐State fMRI Preprocessing Analysis

Minimally preprocessed data were obtained from the HCP database (http://humanconnectome.org). The HCP processed the data through their pipeline, which was specifically designed for high‐quality HCP data. Their data were minimally preprocessed according to Glasser et al. [21]; the preprocessing steps included gradient distortion correction, rigid body realignment, field map processing, spatial normalization to the stereotactic space of the Montreal Neurological Institute (MNI), and independent component analysis‐based denoising [22]. Then, the data were processed using the MATLAB (https://www.mathworks.com/) and DPABI 6.1 [23] toolboxes with the following parameters: (1) bandpass filtering (0.01 and 0.08 Hz), (2) regressing out nuisance signals from white matter and cerebrospinal fluid (CSF) using label maps provided by the HCP organization, and (3) spatial smoothing with a 6 mm full‐width at half maximum (FWHM) Gaussian kernel.

The two CCBD and TCDQ RS‐fMRI datasets were also preprocessed in the DPABI toolbox according to the following steps: (1) slice timing correction was not necessary for the CCBD dataset due to the short repetition time (TR); hence, this step was only performed for TCDQ data; (2) motion correction; (3) spatial normalization to the EPI template and resampling to 3 × 3 × 3 mm^3^; (4) bandpass filtering (0.01 and 0.08 Hz); (5) regressing out nuisance signals, including head motion effects estimated with the Friston 24‐parameters, white matter signals, and CSF signals; and (6) spatial smoothing (FWHM = 6 mm).

### 2.3 Functional Connectivity Calculation

A spherical region of interest (ROI) (radius = 3 mm) was delineated and used as the seed of the left (MNI: −11, −20, 1) and right (MNI: 10, −20, 0) CMs [20]. The mean CM coordinates were provided by Warren et al., who provided a comprehensive account of neurosurgical targeting for CM‐DBS in patients with refractory generalized epilepsy using a novel “edge‐weighted MP2RAGE” image for CM visualization in the individual space and warped into MNI space. In their study, all the CM targets had anatomical locations of intraoperative microelectrode recordings and were defined by the Krauth/Morel thalamic atlas [24]. Moreover, according to version 3 of the Automated Anatomical Labeling (AAL3) atlas [25], the two mean coordinates of the left and right CMs were in the posterior group of the intralaminar thalamus. Next, each participant's blood oxygen level–dependent (BOLD) time series was averaged within each hemisphere of the CM. Thus, for each subject in every group, we obtained an FC map for each seed region, representing the correlation of the activity in each voxel in the brain with the average signal of the specific seed region. Finally, we used Pearson correlation as the metric of association between the time series for voxels across the whole brain with correlation coefficients (*r*). To improve normality, the *r* values were transformed to *Zr* values via Fisher's *r*‐to‐*z* transformation [26].

### 2.4 Group Analysis and Group Cluster Production

**FIGURE 1:**
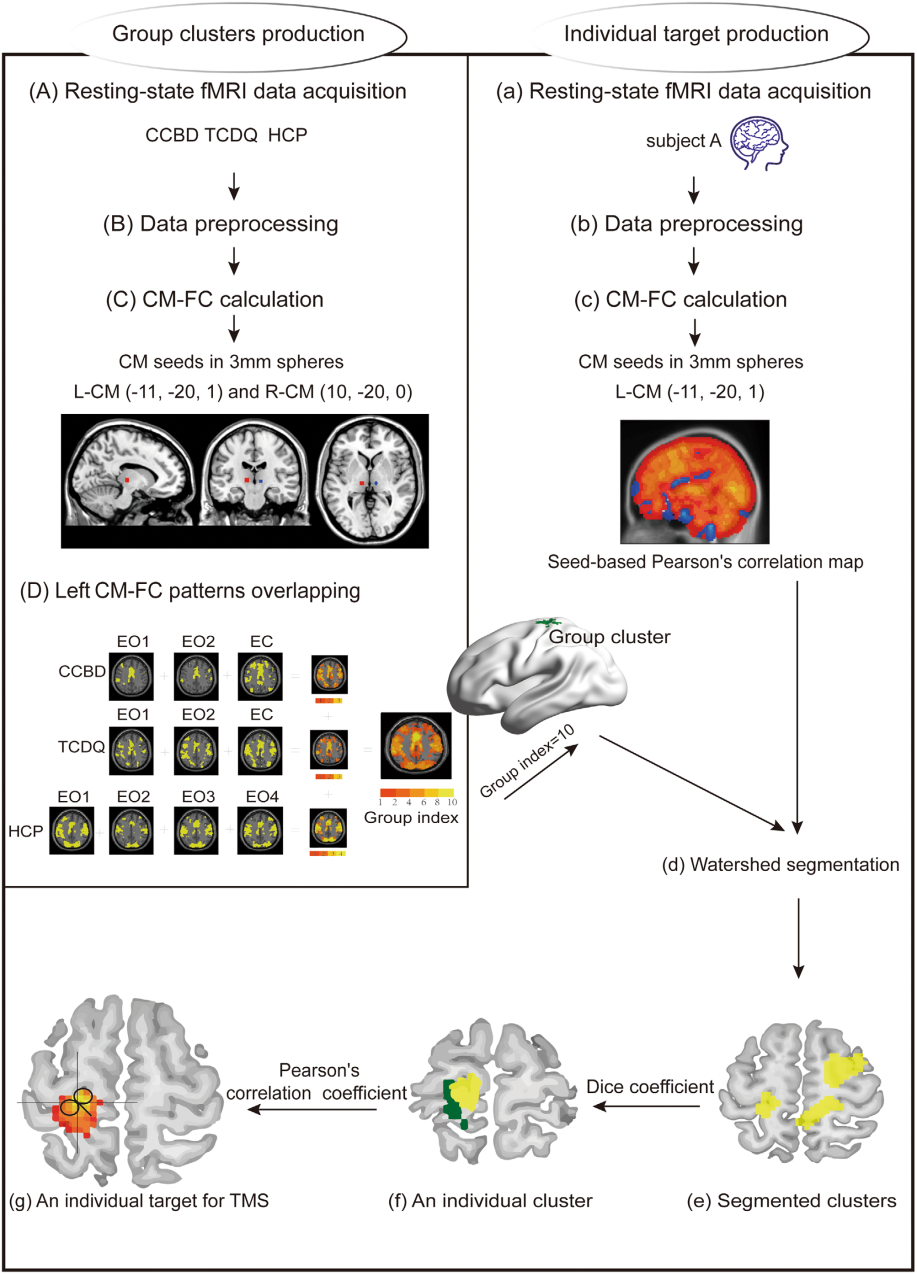
Applied methodological pipeline of the data analysis. This pipeline consists of two parts, the identification of CM‐targeting group clusters (A‐D) and the use of these clusters to localize individual targets for TMS intervention (a‐g). Here, we show an example of CM targeting on the left (c), which is the same application as that of right CM targeting. CCBD, Center of Cognition and Brain Disorders; CM‐FC, centromedian functional connectivity; EC, eyes closed; EO, eyes open; HCP, Human Connectome Project; TCDQ, TMS Center of Deqing Hospital.

The CM‐FC statistical test was performed with a one‐sample *t‐test* of the three datasets (*p* < 0.001 for HCP, *p* < 0.01 for CCBD and TCDQ, uncorrected). For cortical connectivity mapping within a 2‐cm brain mask, none of the CM‐FC maps considered subcortical results. Surviving voxels were marked as 1, and subthreshold voxels were marked as 0 in each group. Then, 10 binary maps from 10 sessions were overlaid (Figure 1D). Finally, we obtained two combined maps, which were labeled left CM‐FC and right CM‐FC, with the “overlapping index” ranging from 0 to 10. Within 4 cm below the scalp, including the 2‐cm brain parenchyma, clusters with more than 30 voxels were chosen as group clusters from the left and right CM. Finally, these two group clusters were identified and used to guide individualized TMS.

### 2.5 Watershed Transformation for Individual Applications

The watershed transform is a popular segmentation method [27]. This method is useful for many different image segmentation applications. In principle, the computation of the watershed transform is based on gradient processing of the original image so that the watershed basinization boundary is located at high gradient points [28, 29]. We used the watershed transform in this study to determine the individual peak coordinates in a cluster that overlapped with the group cluster. More specifically, using two group‐level FC clusters, right CM‐based and left CM‐based, the watershed transformation was used to find the largest region in the individual cluster overlaying the group cluster. The Dice similarity coefficient was calculated by measuring the ratio of overlap between the intersection and sum of the volumes. This index ranges from 0 (no overlap) to 1 (complete overlap). The Dice similarity coefficient is defined as follows:

$$\text{Dice}\left(A,B\right)=\frac{2\left|A\cap B\right|}{\left|A\right|+|B|}$$

Then, in the individual cluster, the cortical peak voxel with the largest *r* value was selected as the individual target for TMS.

Here, we showed an example to explain how this scheme works and prepares for personalized TMS. Firstly, a subject from the CCBD group was randomly selected. After the RS‐fMRI data preprocessing and left CM‐based FC calculation, a map of CM‐FC correlation coefficients (*r*) was obtained (Figure 1a–c). Then, the group cluster of the left CM was used for watershed imaging segmentation (Figure 1d–f). According to the largest Dice index, an individual cluster was identified in Figure 1f. In the left CM‐FC map, the corresponding coordinates of the peak voxel were extracted within the obtained individual cluster in Figure 1g. This peak voxel was taken as the stimulation target for future TMS treatment.

We provided the in‐house script and the group clusters for those who demanded TMS intervention based on the CM as an effective target.

## 3 Results

In this study, CM‐FC analysis was conducted using RS‐fMRI data acquired from 40 healthy participants, divided into three datasets: the HCP, the CCBD, and the TCDQ. Following preprocessing, FC maps were generated using a seed‐based approach, with two ROIs located in the left and right CMs of the thalamus. Group‐level FC statistical maps were produced and combined into an overlapping map across the three datasets. This was followed by individual‐level peak detection using watershed segmentation to identify target sites for precise TMS.

### 3.1 CM‐FC Pattern

**FIGURE 2:**
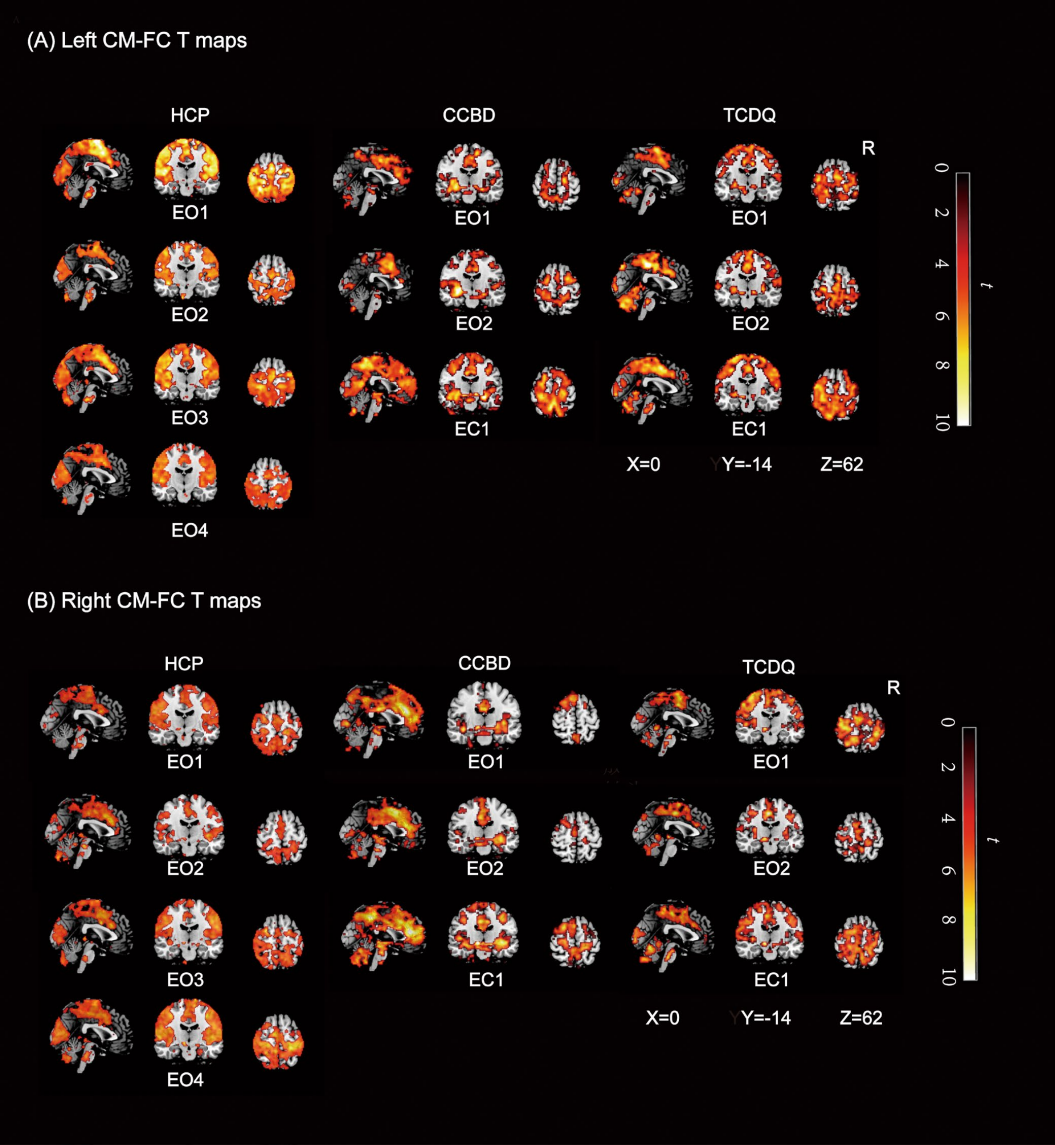
Left CM‐FC and right CM‐FC maps for each session. Sagittal (*X* = 0), coronal (*Y* = −14), and axial (*Z* = 62) brain maps showing areas of significant positive FC strength (*p* < 0.001, uncorrected for HCP and *p* < 0.01, uncorrected for CCBD and TCDQ) calculated separately for the left (A) and right (B) CM in each session from the three MR scanners. The red‐to‐yellow scale represents the statistics (*t*) of the voxelwise analyses testing whether the average connectivity with the left CM (A) and right CM (B) seed regions was statistically significant. The *X*, *Y*, and *Z* coordinates indicate the sagittal, coronal, and axial voxel positions (mm), respectively, in the Montreal Neurological Institute Ch2better template space. CCBD, Center of Cognition and Brain Disorders; CM‐FC, Centromedian functional connectivity; EC, Eyes closed; EO, Eyes open; HCP, Human Connectome Project; TCDQ, TMS Center of Deqing Hospital.

The CM‐FC patterns demonstrated remarkable similarity between the CMs of each hemisphere and across different scanners in the HCP, CCBD, and TCDQ datasets. The similarity in FC patterns across different datasets and scanners highlights the robustness and reproducibility of the observed connectivity. The analysis revealed that both the left and right CMs were functionally connected to several key brain regions. Specifically, strong connections were observed with the middle cingulate gyrus, the supplementary motor area (SMA), the primary sensorimotor cortex (PSMC), the superior temporal gyrus, the superior frontal gyrus (SFG), the cuneus, and the brain stem. Importantly, all these connections exhibited positive correlations, indicating a consistent pattern of synchronous activity between the CMs and these regions. Details are shown in Figure 2 and Table 2.

**TABLE 2:** CM‐based RSFC spatial pattern.

Regions		Peak coordinates			Cluster size	Common index
AAL3	Brodmann	*X*	*Y*	*Z*		
Postcentral_L	BA3_L	−39	−18	42	111	19
Cingulate_Mid_L^a^	BA24_R	9	18	33	1811	20
SupraMarginal_R	BA2_R	60	−30	36	27	16
Frontal_Mid_2_L	BA46_L	−33	39	30	21	16
Calcarine_R (aal)	BA17_R	6	−90	0	41	16
Rolandic_Oper_R^b^	BA38_R	54	9	−3	387	18
Temporal_Sup_L^c^	BA48_L	−45	−27	6	549	20

^a^ There are 59 max points in this cluster.

^b^ There are 7 max points in this cluster.

^c^ There are 2 max points in this cluster.

### 3.2 Group Cluster

**FIGURE 3:**
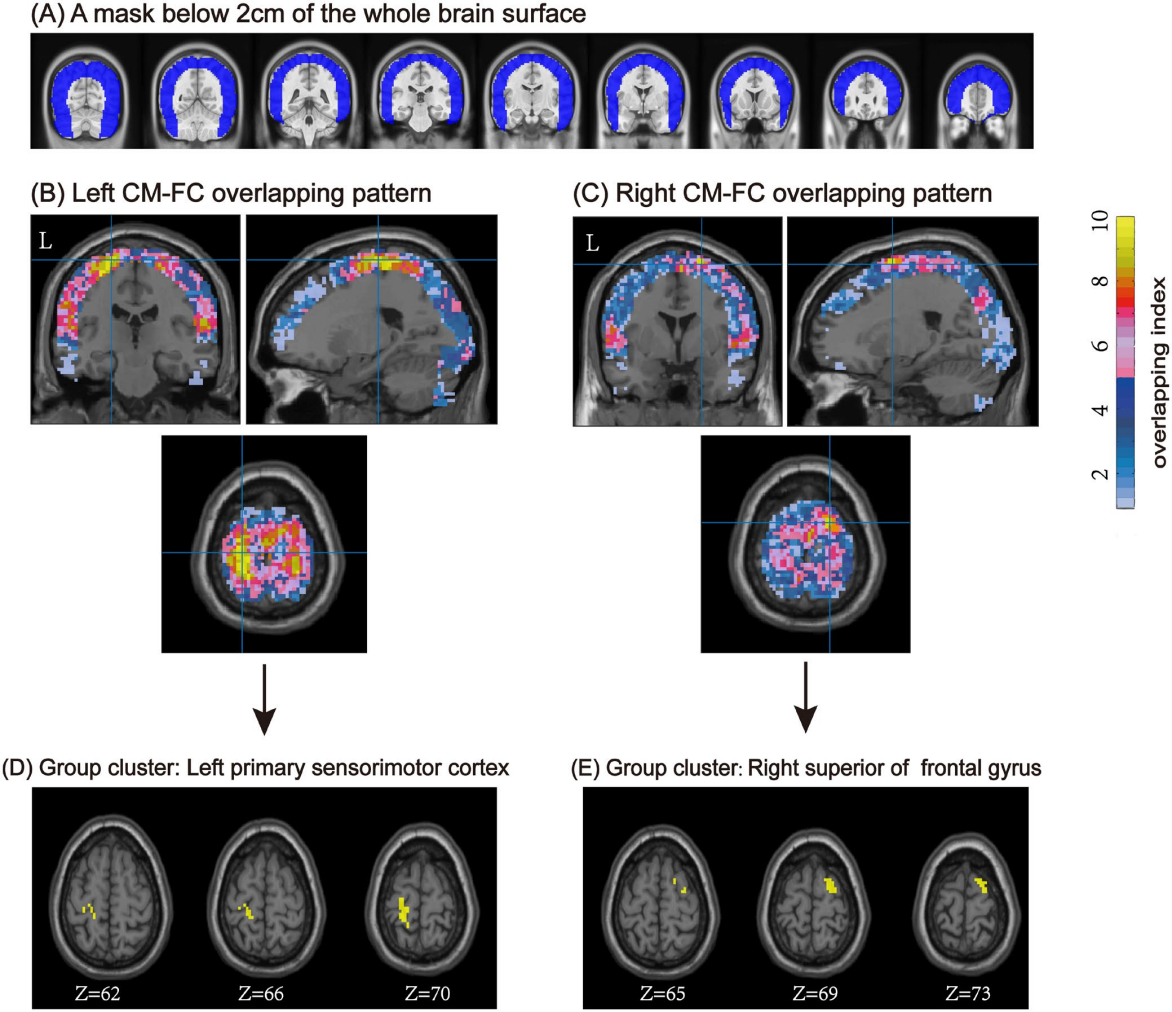
Group CM‐FC pattern and two group clusters. A 2‐cm cortical brain mask was applied to determine the TMS available distance. Spatial connectivity maps for the left CM and right CM in the Ch2 template. The crosshair was located in the voxel within the cluster with a larger overlapping index and more than 30 voxels in each hemisphere. The color bar indicates an overlap index between 0 and 10. The group clusters of the left and right CMs, the left primary sensorimotor cortex, and the right superior frontal gyrus are presented in the three axial panels in the MNI space (thickness = 4 mm).

Our results revealed two distinct group clusters from the left CM‐FC and right CM‐FC overlapping maps. These clusters demonstrated a high degree of overlap, indicating consistent connectivity patterns. Specifically, the overlapping index was significantly common in 10 out of 10 sessions or eight out of 10 sessions, highlighting the reproducibility of the findings across different time points. For the left CM‐FC map, the identified group cluster (10 out of 10 sessions) in the left PSMC comprised 51 voxels, as illustrated in Figure 3D. This cluster represents a robust area of connectivity, emphasizing the left CM's strong functional link with the PSMC, a region crucial for motor control and sensory processing. In the right CM‐FC map, the group cluster (eight out of ten sessions) was located in the right SFG and consisted of 37 voxels, as shown in Figure 3E. This cluster underscores the right CM's connection with the SFG, a region involved in executive functions, decision‐making, and motor planning. This expanded paragraph provides more context and detail about the results.

### 3.3 An Example for an Individual Participant

**FIGURE 4:**
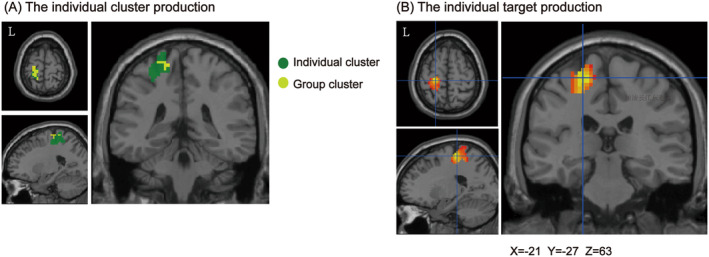
A sample of watershed segmentation in the left CM‐FC. (A) The spatial relationship of the left CM‐based group cluster (left PSMC) and the individual cluster. (B) The crosshair was the peak with the largest *r* value (*r* = 0.237) in the individual cluster (MNI: −21, −27, 63).

The high degree of overlap and consistency in these group clusters (PSMC and SFG) across multiple sessions underscores the reliability of CM‐FC mapping. Based on the map of the subject's CM‐FC correlation coefficients (*r*), an individual cluster (dice index = 0.132) was identified, which showed a spatial most association with the group cluster, PSMC (Figure 4A). This individual cluster's peak correlation coefficient (*r*) was 0.237, and its corresponding location was *X* = −21, *Y* = −27, and *Z* = 63 in MNI space (Figure 4). These coordinates pinpoint the specific individual TMS target within the brain. It is worth noting that the spatial coordinates derived from the MNI space provide exact locations for TMS‐targeted interventions, ensuring that treatments can be tailored to the specific neural architecture of each individual. By ensuring that the individual clusters align well with the group clusters, this scheme may improve the precision and efficacy of TMS treatment.

## 4 Discussion

To our knowledge, we are the first to report CM mapping results across different populations and scanning environments for the TMS treatment of CM‐related neurological diseases. More importantly, we identified two group clusters and the consistency of these clusters supports the use of CM‐FC maps as a stable and dependable tool for identifying precise neuromodulation targets.

### 4.1 CM Seed Localization

The CM is a critical component of the thalamus and has diverse functions related to arousal, attention, sensory processing, and motor control. Given its involvement in a wide variety of functions, dysfunction of the CM has been associated with a range of neurological disorders. In CM‐DBS presurgical planning, the target localization can be identified via three approaches: direct, indirect, and semidirect [30]. The direct approach can be used to visualize the target on conventional MRI sequences without stereotactic coordinates, which is not suitable for the CM because it is a small thalamic region measuring less than 10 mm in every dimension with an approximate volume of 310 mm^3^. The indirect approach using stereotactic coordinates is highly risky for patients with third ventricle dilatation. Although the CM is not visible on conventional imaging, it can be identified on several advanced MRI sequences, such as 3 T proton density‐weighted [24], magnetization‐prepared 2 rapid gradient echo (MP2RAGE) [20], 3D edge‐enhancing gradient echo (EDGE) [31], and quantitative susceptibility mapping (QSM) [32] MRI sequences. Our seeds in the left and right CMs were identified from a prior CM‐DBS study using the MP2RAGE MRI sequence for CM anatomical localization, which reported the active electrode contacts and their coordinates in the MNI space during CM‐DBS mapping for patients with drug‐resistant epilepsy [20]. Then, we checked the two seed locations and found that they were actually located in the posterior group of intralaminar thalamus in the AAL3 [25].

### 4.2 CM Connectivity Mapping

Using resting‐state fMRI sequences from different scanners, our study revealed characteristic CM functional connectivity patterns. Previous TMS‐EEG studies had evidenced that TMS could activate instinct network [33, 34] especially motor network [35], while the lower spatial revolution acquired by scalp EEG limited the TMS precision. Moreover, the CCBD and TCDQ datasets were obtained from the same healthy cohort. To obtain a full picture of the CM pattern, eyes‐open and eyes‐closed scans were both considered in our study, as was the hemisphere of the CM, which was an important condition in the FC analysis [36]. The comprehensive spatial pattern of the CM was profiled and included the middle cingulum, SMA, PSMC, precuneus, supramarginal gyrus, superior temporal lobe, and SFG. The comprehensive CM‐FC patterns were similar to those observed in a previous study [37]. In detail, the connectivity of the CMs with regions involved in motor control, sensory processing, and higher order cognitive functions underscores the central role of the CM in integrating and coordinating complex brain activities. For instance, the connection with the SMA and PSMC suggests a role in motor planning and execution, while the linkage with the superior temporal gyrus and SFG points to involvement in auditory processing and executive functions, respectively. The connection with the cuneus highlights the CM's role in visual processing, and its connectivity with the brain stem emphasizes its involvement in fundamental life‐sustaining functions.

A recent human CM‐DBS site study using diffusion tensor tractography largely confirmed these observations of CM structural connections [38]. Electrophysiological evidence also revealed diffuse electroencephalography (EEG) desynchronization produced by CM stimulation [39]. Given the evidence of widespread connectivity, CM functions in humans are not limited to sensorimotor regulation; the CM is also involved in complex cognitive functions such as attention and consciousness processing. Therefore, it is not surprising that CM modulation has been investigated for the treatment of neurological disorders, such as Tourette syndrome (TS), disorder of consciousness (DoC), and refractory generalized epilepsy (RGE).

### 4.3 Group Clusters: PSMC and SFG

One of our group clusters, the left PSMC, appeared across all scanning sessions (overlapping index = 10) in the left CM‐FC, while the right PSMC did not reach the greatest overlap (overlapping index < 8) in the right CM‐FC. We were unable to determine the reason for this difference, and further studies are needed in the future. According to a previous study, the main function of the PSMC is to initiate movements and to control voluntary motor behavior via the thalamostriate circuit during normal and pathological movements [40]. The CM mainly receives input from parallel motor circuits of the basal ganglia and projects over the entire sensorimotor area of the striatum [41]. This anatomic feature is consistent with our CM‐FC results, indicating that a wide range of sensorimotor areas showed a marked relationship with the bilateral CM. Thus, the CM showed greater functional coupling with the PSMC and provided a motor circuit mechanism for TMS intervention in movement disorders, such as TS. For a better FC effect on motor cortical TMS, Pieramico et al. suggested that the E‐field orientation was a key factor [42] and Tervo et al. proposed an automated closed‐loop TMS–EEG setup to adjust TMS parameters [43]. Optimizing TMS protocols can significantly enhance the impact of CM‐FC on motor cortical areas.

The other group cluster, the SFG, is involved in the modulation of cortical processing during attention‐demanding tasks, suggesting a role for maintaining attention and consciousness [1]. As a core hub in the ascending reticular activation system (ASAS), the CM plays a role in arousal modulation and consciousness maintenance via DBS‐induced overall changes in activity throughout the connected ASAS network [5]. Moreover, CM stimulation may improve awareness and prevent the spread of seizure discharge by disrupting low‐frequency ictal thalamocortical recruitment [44]. The functional characteristics may be a potential mechanism that could be applied in consciousness modulation. Thus, it is possible to utilize the SFG for CM‐targeted recovery of consciousness, such as in DoC.

CM‐DBS has shown satisfactory efficacy in treating RGE [45, 46, 47], which is characterized by loss of consciousness and tonic and myoclonic seizures. Since the CM might control generalized seizures by regulating the excitability of other structures [48, 49], the highly connected PSMC could be modulated by TMS for antiepileptic treatment. In the present study, the TMS site was mapped to underlying CM‐related brain circuits. Our group clusters, PSMC and SFG, showed a high probability of overlap among the three datasets. In an fMRI and diffusion tensor imaging (DTI) network study [37] from Torres Diaz et al., CM‐DBS clinical outcome improvement (> 50%) occurred in 80% of patients with RGE and was closely related to the volume of activated tissue interconnected with the ASAS, encompassing the PSMC and supplementary motor cortices, together with the cerebellum/brainstem. Importantly, they found that CM‐DBS‐related seizure frequency improvement was associated with the number of fibers connecting to the postcentral cortex (*r* = 0.665, *p* = 0.018), precentral cortex (*r* = 0.686, *p* = 0.0.014), and superior frontal cortex (*r* = 0.825, *p* = 0.0017). Therefore, the clinical evidence supported our results, suggesting that the PSMC and SFG also have structural connections with the CM and are related to the efficacy of CM targeting in generalized seizure control.

### 4.4 Limitations

The present study has several limitations. First, the anatomic location of the left and right CM was only identified by MNI coordinates. Thus, other advanced MRI sequences in the individual space and high‐quality atlases are needed in future work. Second, for future patient studies, structural anomalies may affect functional connectivity profiles. Caution should be taken when identifying the individual peak FC as the TMS target. However, given the evidence of high overlap between CM structural and functional connectivity, we can assume that the resulting CM‐based networks are not greatly affected by structural anomalies.

## 5 Conclusion

The left CM of the thalamus showed strong and reproducible FC with the left PSMC. The individual peak FC location in the left PSMC could be considered an individual TMS target for the modulation of CM activity and hence for potential treatment of CM‐related disorders, for example, epilepsy and disorders of consciousness.

## Conflicts of Interest

The authors declare no conflicts of interest.

## Data Availability

The data that support the findings of this study are available from the corresponding author upon reasonable request.

## References

[1] A. Ilyas , D. Pizarro , A. K. Romeo , K. O. Riley , and S. Pati , “The Centromedian Nucleus: Anatomy, Physiology, and Clinical Implications,” Journal of Clinical Neuroscience 63 (2019): 1–7, 10.1016/j.jocn.2019.01.050. 30827880

[2] L. J. Dalic , A. E. L. Warren , K. J. Bulluss , et al., “DBS of Thalamic Centromedian Nucleus for Lennox–Gastaut Syndrome (ESTEL Trial),” Annals of Neurology 91, no. 2 (2022): 253–267, 10.1002/ana.26280. 34877694

[3] P. Bourdillon , B. Hermann , J. D. Sitt , and L. Naccache , “Electromagnetic Brain Stimulation in Patients With Disorders of Consciousness,” Frontiers in Neuroscience 13 (2019): 13, 10.3389/fnins.2019.00223. 30936822 PMC6432925

[4] D. Martinez‐Ramirez , J. Jimenez‐Shahed , J. F. Leckman , et al., “Efficacy and Safety of Deep Brain Stimulation in Tourette Syndrome: The International Tourette Syndrome Deep Brain Stimulation Public Database and Registry,” JAMA Neurology 75, no. 3 (2018): 353–359, 10.1001/jamaneurol.2017.4317. 29340590 PMC5885852

[5] M. Abdallat , A. Saryyeva , C. Blahak , et al., “Centromedian‐Parafascicular and Somatosensory Thalamic Deep Brain Stimulation for Treatment of Chronic Neuropathic Pain: A Contemporary Series of 40 Patients,” Biomedicine 9, no. 7 (2021): 2, 10.3390/biomedicines9070731. PMC8301341 34202202

[6] J. He , H. Zhang , Y. Dang , et al., “Electrophysiological Characteristics of CM‐pf in Diagnosis and Outcome of Patients With Disorders of Consciousness,” Brain Stimulation 16, no. 5 (2023): 1522–1532, 10.1016/j.brs.2023.09.021. 37778457

[7] N. A. Shlobin , K. Hofmann , N. T. Cohen , M. Z. Koubeissi , W. D. Gaillard , and C. O. Oluigbo , “Deep Brain Stimulation of the Centromedian Nucleus of the Thalamus for Lennox‐Gastaut Syndrome: A Systematic Review and Individual Patient Data Analysis,” Neurosurgery 92, no. 4 (2023): 703–715, 10.1227/neu.0000000000002280. 36700706

[8] R. Yang , B. Xiong , M. Wang , et al., “Gamma Knife Surgery and Deep Brain Stimulation of the Centromedian Nucleus for Chronic Pain: A Systematic Review,” Asian Journal of Surgery 46, no. 9 (2023): 3437–3446, 10.1016/j.asjsur.2023.06.026. 37422388

[9] S. Vucic , K. H. Stanley Chen , M. C. Kiernan , et al., “Clinical Diagnostic Utility of Transcranial Magnetic Stimulation in Neurological Disorders. Updated Report of an IFCN Committee,” Clinical Neurophysiology 150 (2023): 131–175, 10.1016/j.clinph.2023.03.010. 37068329 PMC10192339

[10] R. A. Badawy , G. D. Jackson , S. F. Berkovic , and R. A. Macdonell , “Cortical Excitability and Refractory Epilepsy: A Three‐Year Longitudinal Transcranial Magnetic Stimulation Study,” International Journal of Neural Systems 23, no. 1 (2013): 1250030, 10.1142/s012906571250030x. 23273126

[11] V. K. Kimiskidis , A. Valentin , and R. Kälviäinen , “Transcranial Magnetic Stimulation for the Diagnosis and Treatment of Epilepsy,” Current Opinion in Neurology 27, no. 2 (2014): 236–241, https://journals.lww.com/co‐neurology/fulltext/2014/04000/transcranial_magnetic_stimulation_for_the.15.aspx. 24553462 10.1097/WCO.0000000000000071

[12] W. Huang , Q. Chen , J. Liu , et al., “Transcranial Magnetic Stimulation in Disorders of Consciousness: An Update and Perspectives,” Aging and Disease 14, no. 4 (2023): 1171–1183, 10.14336/ad.2022.1114. 37163434 PMC10389824

[13] X. Xia , Y. Bai , Y. Zhou , et al., “Effects of 10 Hz Repetitive Transcranial Magnetic Stimulation of the Left Dorsolateral Prefrontal Cortex in Disorders of Consciousness,” Frontiers in Neurology 8 (2017): 182, 10.3389/fneur.2017.00182. 28515709 PMC5413493

[14] C. K. Kahl , A. Kirton , T. Pringsheim , et al., “Bilateral Transcranial Magnetic Stimulation of the Supplementary Motor Area in Children With Tourette Syndrome,” Developmental Medicine and Child Neurology 63, no. 7 (2021): 808–815, 10.1111/dmcn.14828. 33634500

[15] L. Beynel , J. P. Powers , and L. G. Appelbaum , “Effects of Repetitive Transcranial Magnetic Stimulation on Resting‐State Connectivity: A Systematic Review,” NeuroImage 211 (2020): 116596, 10.1016/j.neuroimage.2020.116596. 32014552 PMC7571509

[16] H. Zhang , N. Sollmann , G. Castrillón , et al., “Intranetwork and Internetwork Effects of Navigated Transcranial Magnetic Stimulation Using Low‐ and High‐Frequency Pulse Application to the Dorsolateral Prefrontal Cortex: A Combined rTMS–fMRI Approach,” Journal of Clinical Neurophysiology 37, no. 2 (2020): 131–139, https://journals.lww.com/clinicalneurophys/Fulltext/2020/03000/Intranetwork_and_Internetwork_Effects_of_Navigated.5.aspx. 30335664 10.1097/WNP.0000000000000528

[17] R. F. H. Cash , A. Weigand , A. Zalesky , et al., “Using Brain Imaging to Improve Spatial Targeting of Transcranial Magnetic Stimulation for Depression,” Biological Psychiatry 90, no. 10 (2021): 689–700, 10.1016/j.biopsych.2020.05.033. 32800379

[18] E. J. Cole , A. L. Phillips , B. S. Bentzley , et al., “Stanford Neuromodulation Therapy (SNT): A Double‐Blind Randomized Controlled Trial,” American Journal of Psychiatry 179, no. 2 (2022): 132–141, 10.1176/appi.ajp.2021.20101429. 34711062

[19] E. J. Cole , K. H. Stimpson , B. S. Bentzley , et al., “Stanford Accelerated Intelligent Neuromodulation Therapy for Treatment‐Resistant Depression,” American Journal of Psychiatry 177, no. 8 (2020): 716–726, 10.1176/appi.ajp.2019.19070720. 32252538

[20] A. E. L. Warren , L. J. Dalic , W. Thevathasan , A. Roten , K. J. Bulluss , and J. Archer , “Targeting the Centromedian Thalamic Nucleus for Deep Brain Stimulation,” Journal of Neurology, Neurosurgery and Psychiatry 91, no. 4 (2020): 339–349, 10.1136/jnnp-2019-322030. 31980515

[21] M. F. Glasser , S. N. Sotiropoulos , J. A. Wilson , et al., “The Minimal Preprocessing Pipelines for the Human Connectome Project,” NeuroImage 80 (2013): 105–124, 10.1016/j.neuroimage.2013.04.127. 23668970 PMC3720813

[22] G. Salimi‐Khorshidi , G. Douaud , C. F. Beckmann , M. F. Glasser , L. Griffanti , and S. M. Smith , “Automatic Denoising of Functional MRI Data: Combining Independent Component Analysis and Hierarchical Fusion of Classifiers,” NeuroImage 90 (2014): 449–468, 10.1016/j.neuroimage.2013.11.046. 24389422 PMC4019210

[23] C. Yan and Y. Zang , “DPARSF: A MATLAB Toolbox for "Pipeline" Data Analysis of Resting‐State fMRI,” Frontiers in Systems Neuroscience 4 (2010): 3, 10.3389/fnsys.2010.00013. 20577591 PMC2889691

[24] A. Krauth , R. Blanc , A. Poveda , D. Jeanmonod , A. Morel , and G. Székely , “A Mean Three‐Dimensional Atlas of the Human Thalamus: Generation From Multiple Histological Data,” NeuroImage 49, no. 3 (2010): 2053–2062, 10.1016/j.neuroimage.2009.10.042. 19853042

[25] E. T. Rolls , C.‐C. Huang , C.‐P. Lin , J. Feng , and M. Joliot , “Automated Anatomical Labelling Atlas 3,” NeuroImage 206 (2020): 116189, 10.1016/j.neuroimage.2019.116189. 31521825

[26] R. Fisher and S. Aylmer , “On the Probable Error of a Coefficient of Correlation Deduced From a Small Sample,” 1921 1, 1–32.

[27] S. Beucher and F. Meyer , “Segmentation: The Watershed Transformation. Mathematical Morphology in Image Processing,” Optical Engineering 34 (1993): 433–481.

[28] V. Grau , A. U. Mewes , M. Alcañiz , R. Kikinis , and S. K. Warfield , “Improved Watershed Transform for Medical Image Segmentation Using Prior Information,” IEEE Transactions on Medical Imaging 23, no. 4 (2004): 447–458, 10.1109/tmi.2004.824224. 15084070

[29] W. Wieclawek , “3D Marker‐Controlled Watershed for Kidney Segmentation in Clinical CT Exams,” Biomedical Engineering Online 17, no. 1 (2018): 26, 10.1186/s12938-018-0456-x. 29482560 PMC5828230

[30] L. G. Remore , M. Omidbeigi , E. Tsolaki , and A. A. Bari , “Deep Brain Stimulation of Thalamic Nuclei for the Treatment of Drug‐Resistant Epilepsy: Are We Confident With the Precise Surgical Target?,” Seizure 105 (2023): 22–28, 10.1016/j.seizure.2023.01.009. 36657225

[31] E. H. Middlebrooks , L. Okromelidze , C. Lin , et al., “Edge‐Enhancing Gradient Echo With Multi‐Image Co‐Registration and Averaging (EDGE‐MICRA) for Targeting Thalamic Centromedian and Parafascicular Nuclei,” Neuroradiology Journal 34, no. 6 (2021): 667–675, 10.1177/19714009211021781. 34121497 PMC8649196

[32] J. Li , Y. Li , L. Gutierrez , et al., “Imaging the Centromedian Thalamic Nucleus Using Quantitative Susceptibility Mapping,” Frontiers in Human Neuroscience 13 (2020): 8, 10.3389/fnhum.2019.00447. PMC6962173 31998098

[33] D. Momi , Z. Wang , and J. D. Griffiths , “TMS‐Evoked Responses Are Driven by Recurrent Large‐Scale Network Dynamics,” eLife 12 (2023): 8, 10.7554/eLife.83232. PMC10121222 37083491

[34] R. A. Ozdemir , E. Tadayon , P. Boucher , et al., “Individualized Perturbation of the Human Connectome Reveals Reproducible Biomarkers of Network Dynamics Relevant to Cognition,” Proceedings of the National Academy of Sciences of the United States of America 117, no. 14 (2020): 8115–8125, 10.1073/pnas.1911240117. 32193345 PMC7149310

[35] L. Marzetti , A. Basti , R. Guidotti , et al., “Exploring Motor Network Connectivity in State‐Dependent Transcranial Magnetic Stimulation: A Proof‐Of‐Concept Study,” Biomedicine 12, no. 5 (2024): 8, 10.3390/biomedicines12050955. PMC11118810 38790917

[36] D. Zumsteg , A. M. Lozano , H. G. Wieser , and R. A. Wennberg , “Cortical Activation With Deep Brain Stimulation of the Anterior Thalamus for Epilepsy,” Clinical Neurophysiology 117, no. 1 (2006): 192–207, 10.1016/j.clinph.2005.09.015. 16364686

[37] C. V. Torres Diaz , G. González‐Escamilla , D. Ciolac , et al., “Network Substrates of Centromedian Nucleus Deep Brain Stimulation in Generalized Pharmacoresistant Epilepsy,” Neurotherapeutics 18, no. 3 (2021): 1665–1677, 10.1007/s13311-021-01057-y. 33904113 PMC8608991

[38] A. E. L. Warren , L. J. Dalic , K. J. Bulluss , B. A. AR , W. Thevathasan , and J. S. Archer , “The Optimal Target and Connectivity for Deep Brain Stimulation in Lennox‐Gastaut Syndrome,” Annals of Neurology 92, no. 1 (2022): 61–74, 10.1002/ana.26368. 35429045 PMC9544037

[39] M. Velasco , F. Velasco , A. L. Velasco , F. Jiménez , F. Brito , and I. Márquez , “Acute and Chronic Electrical Stimulation of the Centromedian Thalamic Nucleus: Modulation of Reticulo‐Cortical Systems and Predictor Factors for Generalized Seizure Control,” Archives of Medical Research 31, no. 3 (2000b): 304–315, 10.1016/s0188-4409(00)00085-0. 11036182

[40] A. J. Baumgartner , J. A. Thompson , D. S. Kern , and S. G. Ojemann , “Novel Targets in Deep Brain Stimulation for Movement Disorders,” Neurosurgical Review 45, no. 4 (2022): 2593–2613, 10.1007/s10143-022-01770-y. 35511309

[41] H. J. Groenewegen and H. W. Berendse , “The Specificity of the ‘Nonspecific’ Midline and Intralaminar Thalamic Nuclei,” Trends in Neurosciences 17, no. 2 (1994): 52–57, 10.1016/0166-2236(94)90074-4. 7512768

[42] G. Pieramico , R. Guidotti , A. E. Nieminen , et al., “TMS‐Induced Modulation of EEG Functional Connectivity Is Affected by the E‐Field Orientation,” Brain Sciences 13, no. 3 (2023): 8, 10.3390/brainsci13030418. PMC10046030 36979228

[43] A. E. Tervo , J. O. Nieminen , P. Lioumis , et al., “Closed‐Loop Optimization of Transcranial Magnetic Stimulation With Electroencephalography Feedback,” Brain Stimulation 15, no. 2 (2022): 523–531, 10.1016/j.brs.2022.01.016. 35337598 PMC8940636

[44] A. Gummadavelli , J. E. Motelow , N. Smith , Q. Zhan , N. D. Schiff , and H. Blumenfeld , “Thalamic Stimulation to Improve Level of Consciousness After Seizures: Evaluation of Electrophysiology and Behavior,” Epilepsia 56, no. 1 (2015): 114–124, 10.1111/epi.12872. 25442843 PMC4689309

[45] A. Valentín , E. García Navarrete , R. Chelvarajah , et al., “Deep Brain Stimulation of the Centromedian Thalamic Nucleus for the Treatment of Generalized and Frontal Epilepsies,” Epilepsia 54, no. 10 (2013): 1823–1833, 10.1111/epi.12352. 24032641

[46] A. L. Velasco , F. Velasco , F. Jiménez , et al., “Neuromodulation of the Centromedian Thalamic Nuclei in the Treatment of Generalized Seizures and the Improvement of the Quality of Life in Patients With Lennox‐Gastaut Syndrome,” Epilepsia 47, no. 7 (2006): 1203–1212, 10.1111/j.1528-1167.2006.00593.x. 16886984

[47] F. Velasco , M. Velasco , F. Jiménez , et al., “Predictors in the Treatment of Difficult‐To‐Control Seizures by Electrical Stimulation of the Centromedian Thalamic Nucleus,” Neurosurgery 47, no. 2 (2000a): 295–304, 10.1097/00006123-200008000-00007. 10942002

[48] J. W. Miller and J. A. Ferrendelli , “The Central Medial Nucleus: Thalamic Site of Seizure Regulation,” Brain Research 508, no. 2 (1990): 297–300, 10.1016/0006-8993(90)90411-4. 2306621

[49] J. W. Miller and J. A. Ferrendelli , “Characterization of GABAergic Seizure Regulation in the Midline Thalamus,” Neuropharmacology 29, no. 7 (1990): 649–655, 10.1016/0028-3908(90)90026-n. 2166925

